# Combined endoscopic transethmoid and transconjunctival en bloc resection of optic nerve tumors in patients lacking functional vision

**DOI:** 10.3389/fonc.2022.975637

**Published:** 2022-10-14

**Authors:** Jieliang Shi, Yunhai Tu, Mingna Xu, Wencan Wu

**Affiliations:** Department of Orbital and Oculoplastic Surgery, Eye Hospital of Wenzhou Medical University, Wenzhou, China

**Keywords:** Novel surgical technique for optic nerve tumors resection Optic nerve tumor, optic nerve sheath meningioma, optic glioma, en bloc resection, endoscopic endonasal surgery

## Abstract

**Background:**

Surgical treatment of optic nerve tumors is challenging. The study’s objective was to evaluate the efficacy of a combined endoscopic transethmoid and transconjunctival approach in patients without functional vision.

**Design:**

A retrospective, noncomparative case series.

**Methods:**

Retrospective data were collected from all patients who had undergone tumor resection using this approach at the authors’ institution between 2015 and 2021. Preoperative assessments included magnetic resonance imaging and ophthalmological examinations, and re-assessments were conducted three months after surgery and regularly during the follow-up period.

**Results:**

Seventeen patients, mean age 35 ± 19.0 years, were enrolled. Of these, 64.7% presented with visual acuity (VA) of light perception or no light perception. Gross total resection was realized in all patients. The average decline in exophthalmos was 3.63 ± 1.93 mm. Tumor histopathological analysis identified 12 optic nerve sheath meningiomas and 5 optic gliomas. The mean follow-up time was 30 months during which there was no local recurrence in any of the patients.

**Conclusions:**

The combined endoscopic transethmoid and transconjunctival approach offers an additional choice for accessing optic nerve tumors. The procedure is both safe and effective and provides an alternative transcranial route to the orbit.

## Background

Gliomas and meningiomas are the most common neoplasms associated with the optic nerve (1). Both types of tumors are often benign and slowly progressive (2, 3). Although the tumors are mostly unilateral (4), they may in some cases extend into the optic canal (OC) and continue intracranially to the optic chiasm (5).

There is controversy over the indications for surgery in patients lacking useful vision (2, 6). Although removal of the tumor will result in complete vision loss in the affected eye, this will halt the intracranial spread of the tumor and thus prevent vision loss in the contralateral eye (7).

There are many surgical approaches for tumors associated with the optic nerve, and include transcranial, transorbital, transcutaneous, and endoscopic transnasal approaches (6–8). The choice of approach is often dictated by the location of the tumor in the orbit and its relationship with the optic nerve (9).

In this study, we describe a consecutive cohort of 17 patients with optic nerve tumors treated at our institution to demonstrate a minimally invasive surgical technique for the removal of the whole tumor in any quadrant.

## Patients and methods

### Patients

The data of all patients treated with optic nerve tumor resection using the combined endoscopic transethmoid and transconjunctival approach between June 2015 and August 2021 were obtained from the medical records of the Eye Hospital of Wenzhou Medical University. Seventeen patients were identified and subsequently included in the study.

Medical records and radiological images were reviewed. Clinical diagnoses were made based on symptoms, signs, and radiology, and were confirmed pathologically. The indication for surgery was loss of vision with or without disfiguring proptosis that was attributable to the tumor (2).

Data on the type of surgery, complications, effects on the cranial nerves, and follow-up outcomes were analyzed. This study was approved by the ethics committee of the Eye Hospital of Wenzhou Medical University, and either the patients or their legal guardians provided written informed consent.

### Surgical technique

**Figure 1:**
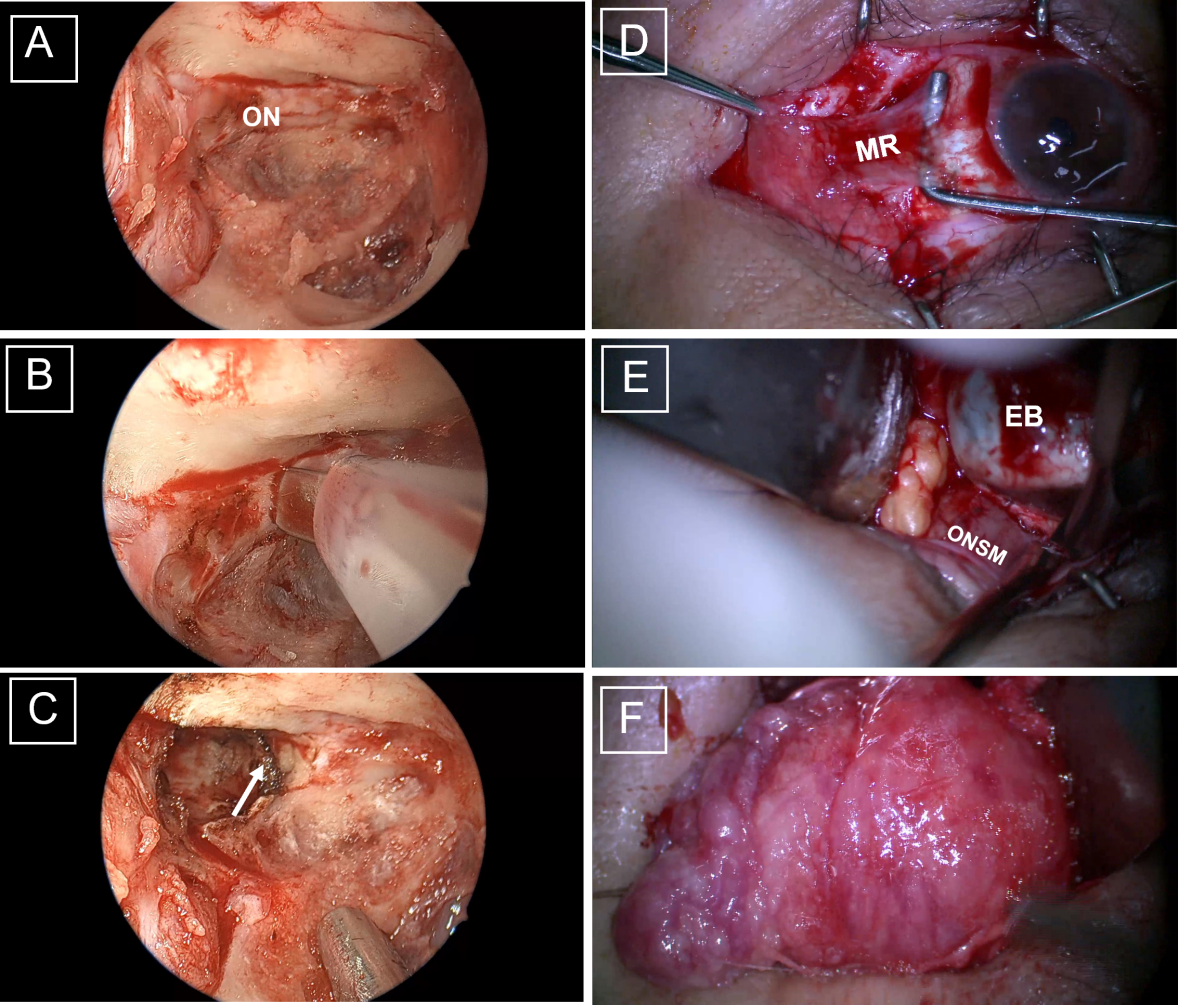
Intraoperative views of the combined endoscopic transethmoid and transconjunctival approach for optic nerve tumor resection. **(A)** Full exposure of the right optic canal. **(B)** The optic nerve was dissected using an electric knife, with the optic nerve section indicated by the white arrow **(C)**. **(D)** After incision of the conjunctiva, the muscle was detached from the bulb according to the position of the muscle. **(E)** The tumor was carefully dissected, with the optic nerve tightly clipped against the eyeball. **(F)** The tumor was gently peeled away from the periorbita. ON, optic nerve; MR, medial rectus muscle; EB, eyeball; ONSM, optic nerve sheath meningioma.

Surgery was conducted under general anesthesia by experienced surgeons. A standard endoscopic sphenoethmoidectomy was carried out using the Messerklinger approach, as detailed in our previous studies (10, 11). The bone was removed up to the orbital apex. The optic protuberance was positioned, and the OC was reduced with a microdrill and removed using a microcurette (**Figure 1A**). The optic nerve was transected at the cranial opening of the OC (12) using an electrosurgical cutting system (ICC 80, ERBE, Germany) (**Figures 1B, C**). The ophthalmic artery was occluded by electrocoagulation at the same time. Great care was taken during the surgery to preserve the integrity of the lateral wall of the optic nerve to prevent accidental cranial tissue injury. In addition, great care was taken to safeguard the arterioles and venules surrounding the eyeball to ensure adequate blood supply to the globe by the collateral branches (13). In the event of cerebrospinal fluid (CSF) leakage, a mucoperiosteal flap with its epithelial side directed to the sinus was used to block the aperture to prevent further seepage of CSF.

The rectus muscle to be isolated was then selected according to the size, location, and morphological characteristics of the tumor being resected. For example, in the case of temporal tumors, the lateral rectus muscle was selected, while in the case of tumors growing nasally, the medial rectus muscle was selected (**Figure 1D**). A relaxing superomedial incision was made in the conjunctiva after which the rectus muscle was isolated and controlled at its insertion into the globe using a double-armed suture. The muscle was separated from the intermuscular septa, and the distal ligaments were separated from the insertion position. Intra-orbital fat was held back with a malleable retractor and the tumor on the optic nerve was visualized after lateral rotation of the globe. Transection of the optic nerve was performed at the site of its attachment to the globe (**Figure 1E**). The tumor was then carefully separated from the surrounding tissue using the transconjunctival approach (**Figure 1F**). The surgical cavity was then thoroughly lavaged with warm saline.

Proptosis of both eyes was evaluated for symmetry. Degradable hemostatic material was used for nasal packing to control bleeding. Finally, the rectus muscle was reconnected at the scleral incision and the conjunctiva was sealed.

### Postoperative examination and follow-up

The postoperative treatment included three days of intravenous methylprednisolone (500 mg) therapy and five days of broad-spectrum antibiotic administration. If CSF leakage was observed during or after surgery, the patient was kept in a bed with an elevated (up to 30°) headrest for seven days. Patients were carefully followed up and exophthalmos and oculomotor activity were closely monitored. A magnetic resonance imaging (MRI) examination was performed three months after the surgery.

## Results

### Clinical features

The demographic and clinical characteristics of the 17 patients are listed in **Table 1**. The mean age of the patients was 35.5 ± 18.9 years (82% of the patients were female). Their presenting VA ranged from 20/1000 to no light perception, with most (64.7%, n=11) being able to only perceive light or nothing at all. The mean onset time was 13.38 ± 8.43 months. All patients presented with relative afferent pupillary defect (RAPD), and other common signs included optic papillary pallor (11 eyes), and ocular motility disorders (8 eyes). Only one patient has ocular pain caused by exposure keratopathy.

**Table 1:** Clinical characteristics of patients.

Case N.	Gender	Age	Duration	Preop VA	Proptosis-Preop.	Proptosis-Postop.	Complications
1	F	40	24	FC/10cm (temperal)	22	18	CSF rhinorrhea
2	F	43	9	HM/10cm	15	13	oculomotor nerve palsy
3	F	54	12	LP	20	16	CSF rhinorrhea& oculomotor nerve palsy
4	F	45	6	HM/BE	17	15	w/o
5	F	42	6	NLP	19	16	w/o
6	F	57	24	LP	20	14	w/o
7	M	43	24	LP	20	17	oculomotor nerve palsy
8	F	52	4	LP	18	16	oculomotor nerve palsy
9	F	52	24	NLP	17	14	w/o
10	F	32	24	HM/20cm	19	13	CSF rhinorrhea
11	F	34	24	NLP	18	NA	w/o
12	F	64	6	LP	16	13	CSF rhinorrhea
13	M	8	0.5	LP	14	13	w/o
14	M	6	12	0.02	18	14	CSF rhinorrhea
15	F	2	4	Lack of directed fixational eye movements	18	14	w/o
16	F	22	12	NLP	24	15	oculomotor nerve palsy
17	F	8	12	HM/BE	16	14	w/o

F, female; M, male; VA, Visual acuity; FC, count fingers; HM, hand motion; NLP, no light perception; LP, light perception; Preop, preoperatively; Postop, postoperatively; CSF, Cerebrospinal fluid; w/o, without.

### Optic nerve tumor resection *via* the combined endoscopic transethmoid and transconjunctival approach

En bloc resection was achieved in 100% of the procedures (see **Supplementary Video**). The median maximal diameter of the tumor was 30 mm (range, 15–50 mm). All tumors were located within the intraconal compartment. The pathological results showed that 70.6% (12/17) of the tumors were primary optic nerve sheath meningiomas (pONSM), while the remaining cases were optic gliomas (5/17). The mean operative time was 2.63 ± 0.62 hours.

### Surgical outcome

All patients presented with identical eye globe proptosis before surgery. Proptosis was resolved in most of the patients. The mean preoperative proptosis was 18.29 ± 2.42 mm and the mean postoperative proptosis was 14.69 ± 1.49 mm, with a mean regression of 3.625 ± 1.93 mm. There was no deterioration in contralateral vision in any of the patients after surgery.

The most frequent complications were intraoperative CSF leakage (5/17) and transient oculomotor nerve palsy (5/17). CSF leakage was fully resolved after strict bed rest. Oculomotor nerve palsy also recovered within one to three months after surgery. No enophthalmos was seen after surgery.

The mean duration of postoperative follow-up was 34 months. None of the patients were treated with postoperative radiotherapy or chemotherapy. No local recurrence or distant tumor metastases was detected in any of the patients during the follow-up time.

### Case illustration

**Figure 2:**
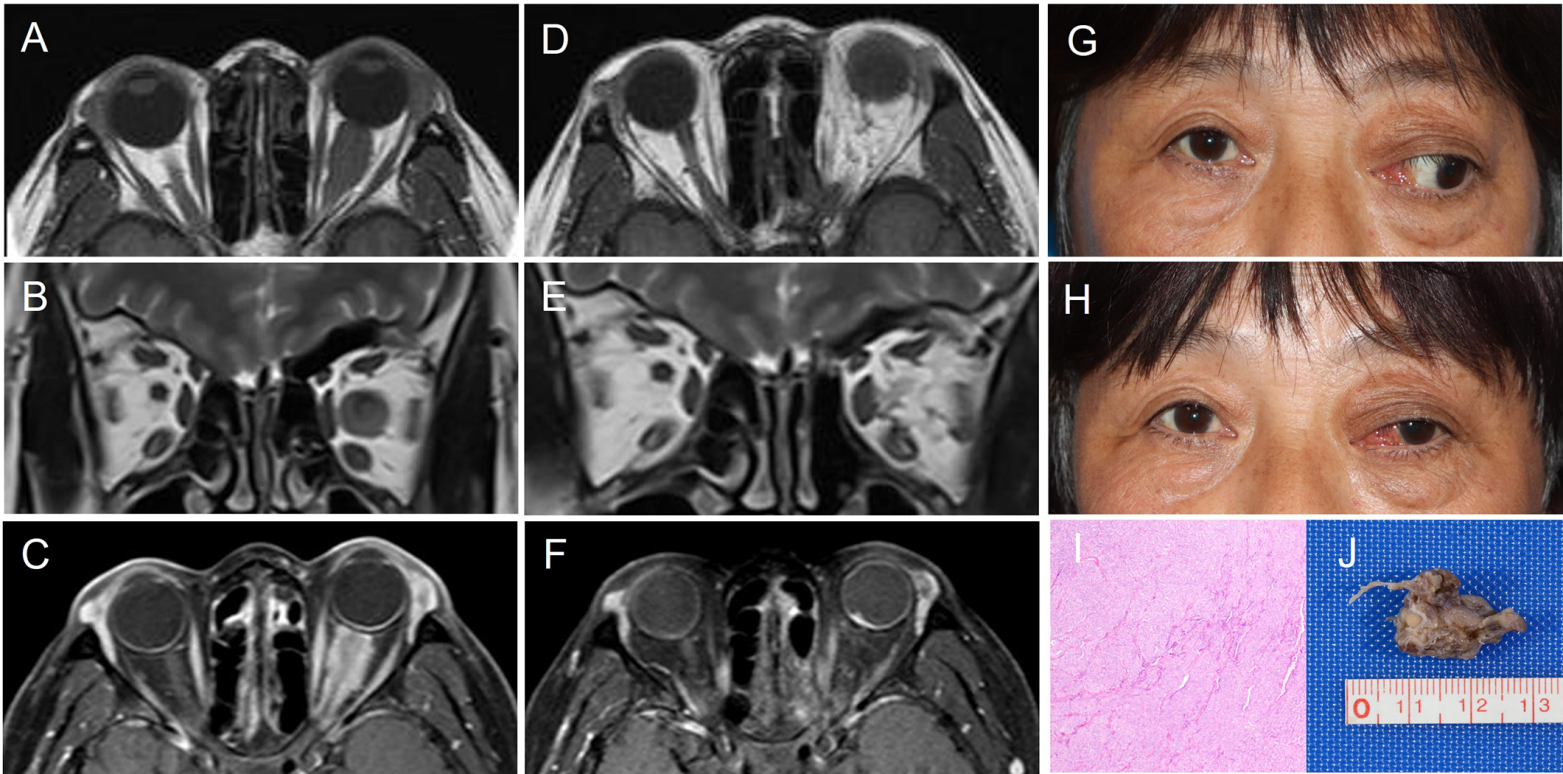
Representative case: a 43-year-old female with longstanding visual loss and proptosis of the left eye. Preoperative MRI, **(A)**T1-weighted axial MRI, **(B)**T2-weighted coronal MRI, **(C)**T1-contrast-enhanced axial MRI. The patient number and corresponding details for this patient (Patient 2) are shown in **Table 1**. Postoperative MRI demonstrating complete tumor removal, **(D)**T1-weighted axial MRI, **(E)**T2-weighted coronal MRI, **(F)**T1-contrast-enhanced axial MRI. **(G, H)** Pre- and postoperative appearance at the one month follow up. Resected tumor pathological findings **(I)** indicated that the tumor was an optic nerve sheath meningioma. The formalin-fixed resected tumor sample **(J)**.

One case is illustrated below (**Figure 2**). A 43-year-old female presented with proptosis of the left eye for nine months with progressively decreasing VA for five months. She had HM/10cm in the left eye and normal VA in the right; the left pupil diameter was 5 mm, RAPD (+), with proptosis and papilledema in the left eye, but otherwise intact. The MRI findings showed that the tumor was pONSM with its origin in the optic nerve. Postoperative MRI confirmed that the tumor had been completely resected. Pathological investigation verified the diagnosis of optic nerve sheath meningioma. The patient recovered well and showed no tumor recurrence at the 13-month follow-up.

## Discussion

There is still controversy over the optimal treatment regimen for pONSM and optic pathway glioma (OPG) without functional vision (14). Previous studies have shown that radiotherapy may have some efficacy in treating these tumors. However, radiotherapy may aggravate adhesion of the tumor to the surrounding tissue, making total resection more difficult, as well as increasing the likelihood of injuring a neighboring vital structure during the surgery (15). Radiotherapy should also be used with caution in pediatric patients, given the burden of adverse effects on neurocognitive and hypothalamic function (6).

For patients with severe visual loss, we performed total resection of the intraorbital and intracanalicular optic nerve together with the tumor. Cristante et al. (16) have suggested that patients with poor VA are unlikely to benefit from debulking surgery because the visual outcome is correlated with preoperative vision loss; thus, the worse the preoperative vision, the less likely that the vision will improve postoperatively (17). In addition, studies have shown that postoperative improvements in vision are correlated with the duration of the preoperative symptoms (18), and that improved postoperative vision was only seen in patients who had experienced a rapid decline in vision (<5 months before surgery) (19). In the present study, all the patients had long mean onset times (13 months). Since subtotal resection is highly prone to tumor recurrence (20), a second operation or postoperative radiotherapy may be required. OPG arises from astrocytes supporting the optic nerve, while pONSM arises from cap cells in the arachnoid surrounding the optic nerve (13); therefore, it is very unusual to achieve total tumor resection without jeopardizing vision. Complete tumor resection could be beneficial not only for the reduced risk of recurrence (21) but also for the prevention of contralateral vision loss (22, 23).

Traditionally, ONSM and OPG tumors have been primarily treated *via* craniotomy, as the transcranial approach provides a clear operative field and enables the removal of tumor tissue within the brain (20, 24). However, this procedure inevitably results in prolonged operative times and hospitalization, together with the potential risk of significant complications and brain tissue damage in some cases. These risks have led clinicians to seek ways of reducing the invasiveness of the procedure where possible, for example, by the removal of optic nerve tumors *via* nasal endoscopy (8) and a lateral orbital approach. While both of these approaches can effectively reduce both the operative time and length of hospitalization, the lateral orbital approach cannot achieve complete resection of intracanalicular tumors and usually results in a substantial cutaneous surgical scar (7), while nasal endoscopy alone is not well-suited to the removal of tumors growing laterally to the temporal side (9) and also increases the medical expenditure on the reconstruction of the orbital wall (25).

The first strength of the approach used in this study lies in the removal of the tumor *via* a transconjunctival approach rather than the dissection of the tumor within the enlarged intermuscular space (25, 26), thus avoiding the ablation of the lamina papyracea and its associated complications. The application of the 360-degree “round-the-clock” approach to accessing the orbit (9, 27) has led to the proposal of the endoscopic endonasal approach for lesions in the mid, posterior, or apical orbital regions from 1 to 7 o’clock. In the present study, resection of the tumor located in either direction could be achieved simply by isolating the corresponding rectus muscle. In general, the transconjunctival approach does not require an osteotomy (28, 29), and exposure of tumors situated near the orbital apex may be restricted by the intraorbital soft tissues (9). The endoscopic transethmoidal-sphenoidal approach has substantial advantages over both the transcranial and purely transconjunctival approaches for the resection of tumors in the optical apex or intracanalicular segment of the optic nerve. Shriver et al. (22) have suggested that gliomas involving the intracanalicular segment of the optic nerve require the use of the transcranial approach for total resection. However, nasal endoscopic surgery has the advantages of a wide surgical field, multiple angles, and strong light conductivity, allowing it to be carried out under direct vision (11, 27). In this study, we did not observe any tumor recurrence during the follow-up period, indicating the effectiveness of complete en bloc tumor removal, regardless of the tumor size. An en bloc resection rather than “in block” fashion (27) can be effective to avoid tumor seeding and recurrence and be more conducive to long-term survival (22, 30).

The combined endoscopic transethmoid and transconjunctival approach is applicable for patients with severe vision loss or disfiguring proptosis with exposure keratopathy and is effective in preventing tumor spread to the contralateral nerve and the risk of bilateral vision loss. In the present cases, although complete tumor removal inevitably resulted in vision loss on the affected side, gross total resection was realized in all patients, with no subsequent tumor recurrence or death in any of the patients. Our preliminary results indicated that the patients did not experience abnormal eye movements, sunken eyeballs, eyeball atrophy, or skin scarring, and the overall result was aesthetically pleasing (27). However, the patients experienced a certain amount of temporary reduction in extraocular movement after the surgery, possibly resulting from mechanical or vascular trauma to the cranial nerve.

## Limitations

There are, nevertheless, several limitations to this approach. The procedure described herein is not suited to the removal of optic nerve tumors involving intracranial regions of the optic nerve. Moreover, this procedure requires surgeons to be skilled in nasal endoscopy, thus limiting more widespread dissemination and implementation of the technique. In addition, this was a relatively small case series with an insufficiently long follow-up, and additional large-scale studies will be essential to establish the long-term efficacy of this approach.

## Conclusions

In summary, the combined endoscopic transethmoid and transconjunctival en bloc resection approach described herein offers a surgical alternative for treating optic nerve tumors in patients lacking functional vision that reduces surgical risk and enhances operative efficiency. However, as the study included a relatively small sample size, final conclusions can only be drawn after further verification with larger numbers of cases.

## Data availability statement

The raw data supporting the conclusions of this article will be made available by the corresponding author on reasonable request. Requests to access the datasets should be directed to ysgxsjl@126.com.

## Ethics statement

The studies involving human participants were reviewed and approved by Ethics Committee of The Eye Hospital of Wenzhou Medical University. Written informed consent to participate in this study was provided by the participants’ legal guardian/next of kin. Written informed consent was obtained from the individual(s) for the publication of any potentially identifiable images or data included in this article.

## Author contributions

All authors participated in planning the study. JS and W-CW participated in drafting the article or critically reviewing the article for important intellectual content. YT and MX participated in analysis and interpretation of data. All authors contributed to the article and approved the submitted version.

## Funding

This study was supported by the National Key Research and Development Program of Zhejiang Province (2019C03009) and the Key Research and Development Program of Wenzhou Eye Hospital (YNZD1201902). The sponsor or funding organization had no role in the design or conduct of this research. All authors had no conflicts of interest to declare.

## Acknowledgment

The authors thank Dr. Guangming Zhou and Ende Wu for providing medical care for the patients.

## Conflict of interest

The authors declare that the research was conducted in the absence of any commercial or financial relationships that could be construed as a potential conflict of interest.

## Publisher’s note

All claims expressed in this article are solely those of the authors and do not necessarily represent those of their affiliated organizations, or those of the publisher, the editors and the reviewers. Any product that may be evaluated in this article, or claim that may be made by its manufacturer, is not guaranteed or endorsed by the publisher.

## Abbreviations

VA, Visual Acuity; OC, Optic Canal; CSF, Cerebrospinal Fluid; RAPD, Relative Afferent Pupillary Defect; pONSM, primary Optic Nerve Sheath Meningiomas; OPG, Optic Pathway Glioma.
